# CD30 and ALK combination therapy has high therapeutic potency in *RANBP2-ALK-*rearranged epithelioid inflammatory myofibroblastic sarcoma

**DOI:** 10.1038/s41416-020-0996-2

**Published:** 2020-07-20

**Authors:** Ashleigh M. Fordham, Jinhan Xie, Andrew J. Gifford, Carol Wadham, Lisa T. Morgan, Emily V. A. Mould, Mitali Fadia, Lei Zhai, Hassina Massudi, Zara S. Ali, Glenn M. Marshall, Robyn E. Lukeis, Jamie I. Fletcher, Karen L. MacKenzie, Toby N. Trahair

**Affiliations:** 1 Children’s Cancer Institute, Lowy Cancer Research Centre, UNSW Sydney, Kensington, NSW Australia; 2 Department of Anatomical Pathology, Prince of Wales Hospital Randwick, Randwick, NSW Australia; 3 ACT Pathology, The Canberra Hospital, Garran, ACT Australia; 4 Children’s Medical Research Institute, Westmead, NSW Australia; 5 Kids Cancer Centre, Sydney Children’s Hospital, Randwick, NSW Australia; 6 School of Women’s and Children’s Health, UNSW Sydney, Kensington, NSW Australia; 7 Cytogenetics Laboratory, SydPath, St Vincent’s Hospital, Darlinghurst, NSW Australia; 8 Faculty of Medicine and Health, University of Sydney, Camperdown, NSW Australia

**Keywords:** Cancer models, Targeted therapies, Sarcoma, Paediatric cancer, Cancer models

## Abstract

**Background:**

Epithelioid inflammatory myofibroblastic sarcoma (eIMS) is characterised by perinuclear ALK localisation, CD30 expression and early relapse despite crizotinib treatment. We aimed to identify therapies to prevent and/or treat ALK inhibitor resistance.

**Methods:**

Malignant ascites, from an eIMS patient at diagnosis and following multiple relapses, were used to generate matched diagnosis and relapse xenografts.

**Results:**

Xenografts were validated by confirmation of *RANBP2-ALK* rearrangement, perinuclear ALK localisation and CD30 expression. Although brentuximab-vedotin (BV) demonstrated single-agent activity, tumours regrew during BV therapy. BV resistance was associated with reduced CD30 expression and induction of ABCB1. BV resistance was reversed in vitro by tariquidar, but combination BV and tariquidar treatment only briefly slowed xenograft growth compared with BV alone. Combining BV with either crizotinib or ceritinib resulted in marked tumour shrinkage in both xenograft models, and resulted in prolonged tumour-free survival in the diagnosis compared with the relapse xenograft.

**Conclusions:**

CD30 is a therapeutic target in eIMS. BV efficacy is limited by the rapid emergence of resistance. Prolonged survival with combination ALK and CD30-targeted-therapy in the diagnosis model provides the rationale to trial this combination in eIMS patients at diagnosis. This combination could also be considered for other CD30-positive, *ALK*-rearranged malignancies.

## Background

Inflammatory myofibroblastic tumour (IMT) is a rare soft tissue sarcoma comprised of myofibroblastic spindle cells and an accompanying inflammatory infiltrate.^1–5^ Chromosomal translocations resulting in fusion of the anaplastic lymphoma kinase gene (*ALK*) with a variety of partner genes occur in ~50% of IMTs.^6,7^ *RAN Binding Protein 2-ALK (RANBP2-ALK)* rearranged epithelioid inflammatory myofibroblastic sarcoma (eIMS) is a clinically aggressive variant of IMT characterised by epithelioid tumour cell morphology, perinuclear ALK staining, CD30 expression and early relapse despite crizotinib treatment.^8–10^ Additional therapeutic options are needed to prevent relapse and/or treat recurrent disease.

Surgical resection remains the preferred method of treatment for localised IMT.^10,11^ However, with the identification of *ALK* fusions, ALK inhibitors (ALKi), including crizotinib, have been used for the treatment of *ALK*-rearranged IMT.^10,12–14^ Crizotinib is an effective ATP-competitive ALK inhibitor with activity in *ALK*-rearranged cancers, including anaplastic large-cell lymphoma (ALCL) and non-small-cell lung cancer (NSCLC).^15–19^ Dramatic and durable responses to crizotinib have been observed in ALK-positive IMT patients.^10,12,20^ Disease relapse during ALKi treatment occurs in *ALK*-rearranged NSCLC and eIMS.^10,21–24^ In our recent retrospective analysis of eight patients with *ALK*-positive IMT, including three with eIMS, we observed that surgery and crizotinib were effective in managing patients with multifocal ALK-positive IMT, with most patients being able to cease crizotinib therapy.^10^ In contrast, two of three patients with *RANBP2-ALK-*rearranged eIMS experienced early disease recurrence despite initial responses to crizotinib.^10^

CD30 expression is a characteristic pathologic feature of eIMS,^8,10,25–28^ which represents a potential therapeutic target using the CD30-targeted antibody–drug conjugate brentuximab vedotin (BV).^29^ BV comprises a monoclonal CD30 antibody, conjugated to the antimitotic agent monomethyl auristatin-E (MMAE),^29^ and is currently used in the treatment of patients with Hodgkin's lymphoma and ALCL.^29–31^ We hypothesised that BV or a combination of BV and ALKi may be effective in treating eIMS. Herein, we report the development of a matched pair of diagnosis and relapse xenograft models established from a patient with *RANBP2-ALK*-rearranged eIMS.^9,10^ Using these models, we demonstrate that CD30 is a therapeutic target in eIMS and the efficacy of combination therapy targeting both CD30 and ALK. These combinations have the potential to prevent relapse or treat ALKi-resistant eIMS and could also be considered for other CD30-positive, *ALK*-rearranged malignancies including ALCL.

## Methods

### Collection of eIMS patient samples

**Fig. 1:**
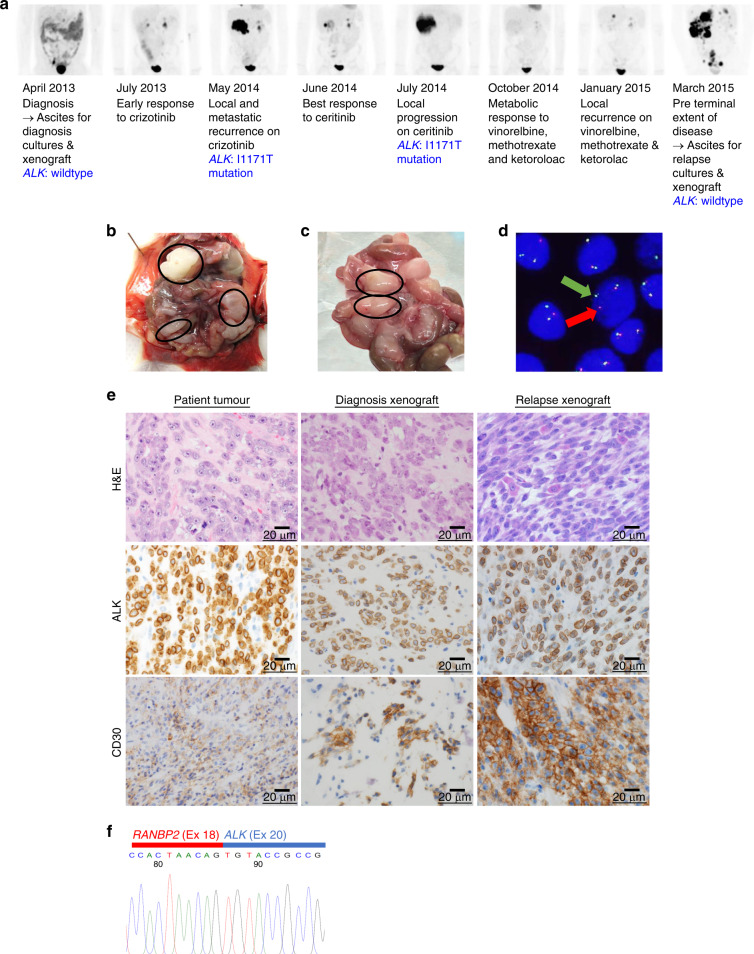
eIMS xenografts recapitulate the features of the patient’s disease. **a** The patient’s clinical response to successive treatments measured by FDG–PET scan and timepoints where patient samples were collected. The identification of the ALK-resistance mutation has been previously reported;^9^ xenografts were established by intraperitoneal injection of eIMS cells from cultured ascites harvested at **b** diagnosis and **c** relapse. Multinodular disease developed in the abdominal cavity of each inoculated mouse. Tumour nodules are circled. **d** Cell suspensions and short-term cultures of xenograft cells were analysed by FISH using a break-apart ALK locus probe. *ALK* gene rearrangement is indicated by the split signal (indicated by red and green arrows). A representative image of diagnosis xenograft cells shown. **e** Xenograft tumours retain histopathologic features of the patient tumour, including perinuclear ALK localisation and CD30 expression. Images ×600 magnification. **f** Sanger sequencing of xenograft RNA confirming an in-frame *RANBP2-ALK* fusion between exon 18 (Ex 18, red bar) of *RANBP2* and exon 20 (Ex 20, blue bar).

This study was approved by the Sydney Children’s Hospital Network Human Research Ethics Committee (LNR/14/SCHN/90) and informed consent was obtained from the patients’ parents. The eIMS biospecimens were collected from a patient (Male, 9.1 years of age)^10^ treated at the Kids Cancer Centre, Sydney Children’s Hospital (Fig. 1a). The patient experienced multiple relapses despite sequential treatment with crizotinib, ceritinib and chemotherapy. The patient died of progressive cancer.^9,10^

### Cell culture

eIMS cells were cultured from malignant ascites samples in flasks coated with 0.1% gelatine (Sigma-Aldrich) in Alpha minimum essential media (αMEM) (Invitrogen) plus 20% foetal bovine serum (FBS) (Life Technologies) in humidified incubators with 5% O_2_ and 5% CO_2_. eIMS samples were validated against patient germline DNA derived from peripheral blood by short tandem repeat (STR) profiling at the Garvan Institute for Medical Research or CellBank Australia (Table 1). MRC-5 human myofibroblasts were purchased from the American Type Culture Collection (ATCC; Rockville, MD, USA) and Karpas299 cells were acquired from the European Collection of Authenticated Cell Cultures via Sigma-Aldrich. These cells were cultured according to the product data sheets.

**Table 1:** STR profiling confirms identity of eIMS cell and xenograft models to patient germline material.

	Germline	Diagnosis cells	Diagnosis xenograft	Relapse cells	Relapse xenograft
D5S818	12	12	12	12	12
D13S317	11, 14	11, 14	11, 14	11, 14	11, 14
D7S820	9, 11	9, 12	9, 12	9, 11	9, 11
D16S539	9, 13	9, 13	9, 13	9, 13	9, 13
vWA	16,17	17	17	17	17
TH01	3, 4, 9	6, 8	6, 8	6, 8	6, 8
Amel	X, Y	X, Y	X, Y	X, Y	X, Y
TPOX	8	8	8	8	8
CSF1PO	10, 11	10, 11	10, 11	10, 11	10, 11
D21S11	29, 31	29, 31	29, 31	29, 31	29, 31
D3S1358	15,16	15, 16	15, 16	15, 16	15, 16, 17
D18S51	14, 17	14, 17	14, 17	14, 17	14, 17
FGA	22, 24	22, 24	22, 24	22, 24	22, 24
Penta E	10, 12	10, 12	10,12	10, 12	10, 12
Penta D	9, 10	9,10	9, 10	9, OL	9, 10
% Match		84.9	84.9	84.9	88

eIMS cells derived from patient ascites and xenograft tumours were validated against patient germline DNA derived from peripheral blood by STR profiling at the Garvan Institute for Medical Research or CellBank Australia.

### Establishment of eIMS xenografts

All animal procedures performed in this study were approved under UNSW Animal Care and Ethics Committee (14/112B, 16/105B and 17/101B). Five to nine-week-old female non-obese diabetic/severe combined immunodeficiency/interleukin 2 receptor gamma (null) (NSG) mice^32,33^ were purchased from Australian BioResources (Moss Vale, NSW, Australia.) and allowed to acclimatise for one week in the pathogen-free environment at the Children’s Cancer Institute. For all inoculations, mice were anaesthetised using 4% isoflurane (Isothesia, ProVet^®^, Eastern Creek, NSW, Australia, Cat#ISOF00) administered by a gaseous anaesthetic machine (Stinger Streamline Rodent/Exotics Anaesthetic Gas Machine, AAS Research and Development, Gladesville, NSW, Australia) while resting on a heated mat. Mice were subjected to subcutaneous injection of 5 × 10^6^ tumour cells suspended in 50% Matrigel (In Vitro Technologies) in αMEM, or intraperitoneal injection of 5 × 10^6^ tumour cells in αMEM. For tertiary passages of the diagnosis xenograft, tumour pieces approximately 3 mm^3^ were implanted subcutaneously according to previously published protocols.^34^ For all in vivo studies, monitoring occurred according to University of New South Wales Animal Care and Ethics Committee approvals. Mice were inspected daily by Children’s Cancer Institute Animal Facility staff for general well-being and weighed twice weekly. Ethical endpoints were defined as a tumour volume of 1000 mm^3^ or 20% loss of original body weight. An experimental endpoint was also defined for mice with no evidence of tumour regrowth 170 days after the cessation of treatment. Mice were euthanised by asphyxiation with CO_2_ followed by cervical dislocation, away from other animals.

### Tumour histology

Excised xenograft tumours were subjected to histologic and immunohistochemical (IHC) analyses to verify eIMS pathology and marker expression. Fixation, embedding and slide preparation was performed at the Garvan Institute for Medical Research Histopathology Facility. Immunohistochemical staining was performed at Anatomical Pathology, South East Area Laboratory Services, Prince of Wales Hospital or Garvan Histopathology Facility using ALK-1 (Leica Biosystems, Cat#ALK-L-CE-H, 1:100) or CD30 (Abacus ALS, Cat#CM130M95 1:300) antibodies. CD30 expression was assessed in ten fields of view by two independent, blinded investigators using a semi-quantitative scoring system that took into account both the intensity of staining and proportion of CD30-positive tissue.^35^

### Xenograft tumour dissociation

To derive single-cell suspensions from xenograft tumours, tumour pieces (~500 mm^3^) were dissociated using the MACs Human Tumor Dissociation Kit (Miltenyi Biotech) according to the manufacturer’s instructions for ‘Dissociation of Medium Tumors with Depletion of Red Blood Cells’ on the gentleMACS Octo Dissociator program 37C_h_TDK_2. The resulting cell suspension was subject to mouse cell depletion using the Mouse Cell Depletion Kit on the autoMACS® Pro Separator program Dpls_7 and cryopreserved or utilised in flow cytometry or in vitro growth-inhibition assays.

### Flow cytometry

For assessment of cell surface CD30, H2-K1 or HLA expression on eIMS cell suspensions, cells were suspended in 100 µL phosphate-buffered saline (PBS) (Sigma-Aldrich) with 2% FBS and incubated with 20 µL of phycoerythrin (PE) conjugated anti-CD30, 5 µL of PE conjugated anti-H2-K1 or 5 µL of allophycocyanin (APC) conjugated anti-HLA (BD Biosciences) for 30 min at 4 °C. Excess antibodies were removed by washing with 500 µL of cold PBS with 2% FBS prior to analysis. Cells were acquired on a FACsCantoII (BD Biosciences) machine, and post-procedural analyses performed using FlowJo software (FlowJo LCC).

### ALK fluorescence in situ hybridisation

Cultures at 50% confluence (~80 × 10^5^ cells) were subject to fluorescence in situ hybridisation (FISH) using the ALK break-apart translocation probe (ZytoLight ® SPEC ALK Dual Color Break Apart Probe, ZytoVision Cat#2124-200)^36^ at the Cytogenetics Laboratory, SydPath, St Vincent’s Hospital.

### *RANBP2-ALK*-fusion validation

RNA was extracted using the AllPrep® DNA/RNA/Protein Mini Kit (QIAGEN) or the RNeasy Plus Mini Kit (QIAGEN). cDNA was synthesised using the SuperScript™ III Reverse Transcriptase Kit according to the manufacturer’s instructions. PCR amplification across the *RANBP2-ALK* fusion was performed using the forward primer: 5′ GCAGTAACTCAGCATCCCCTC and the reverse primer 5′ CAGCAAAGCAGTAGTTGGGG (Sigma-Aldrich) with the AmpliTaq Gold™ DNA Polymerase kit (Thermo Fisher Scientific) on a Veriti® Thermal Cycler (ThermoFisher Scientific) with an initial 95 °C 5 min cycle, followed by 10 cycles at: 94 °C for 30 s, 65 °C for 45 s with a delta down of 1 °C per cycle, 72 °C for 1 min, followed by 25 cycles of 94 °C for 30 s, 55 °C for 45 s, 72 °C for 1 min, followed by 72 °C for 10 min and 4 °C infinite. Sanger sequencing was performed at the Ramaciotti Centre for Functional Genomics, UNSW, Sydney, using the same primers. For the xenografts only, PCR products, generated with the primers 5′ CTCGATGGGCAGAAGATCAG (forward) and 5′ CCTGGCCTTCATACACCTCC (reverse) were ligated into the pGEM®-T Easy Vector System (Promega) and resultant transformants were screened for inserts by restriction enzyme digestion. Sanger sequencing was performed at the Ramaciotti Centre for Functional Genomics, UNSW Sydney Australia and AGRF Melbourne Australia using commercial pUC/M13 sequencing primers.

### Western blot

Protein extraction was performed with the AllPrep® DNA/RNA/Protein Mini Kit (QIAGEN) according to the manufacturer’s instructions. Proteins were separated on Criterion Midi Gels (Bio-Rad Laboratories) in 25 mM Tris base (Univar), 190 mM glycine (Univar) and 0.1% sodium dodecyl sulphate (Sigma-Aldrich) in MilliQ water, pH 8.3 buffer. Proteins were transferred onto nitrocellulose membranes (GE Protran) using the Criterion Blotter transfer tank (Bio-Rad Laboratories) in a 25 mM Tris base, 190 mM glycine, and 20% methanol (Univar) buffer. The membranes were incubated with the following primary antibodies diluted in 5% bovine serum albumin (BSA) (Life Technologies) in Tris base sodium chloride (Univar) with 0.1% Tween-20 (Sigma-Aldrich) (TBST) and incubated overnight at 4 °C: anti-P glycoprotein (ABCB1) antibody ([EPR10364-57], Abcam, Cat#ab170904) at 1:500, and GAPDH antibody (Santa Cruz Biotechnology, Cat#sc-47724) at 1:1000. Secondary goat anti-rabbit (Life Technologies, Cat# 32460) and goat anti-mouse (Life Technologies, Cat#32430) were diluted 1:1000 in 5% BSA in TBST and incubated at room temperature for 1 h. Signals were visualised and quantified on the Bio-Rad ChemiDoc Touch System (Bio-Rad Laboratories) using Image Lab software v5.2.1 (Bio-Rad Laboratories).^37^

### In vitro growth-inhibition assays

For growth-inhibition assays, cells were seeded into 96-well plates (Sigma-Aldrich) in 50 µL of normal culture medium. MRC-5 and eIMS cells were seeded at 2.0 × 10^3^ cells/well and Karpas299 cells at 2.5 × 10^4^ cells/well. Serial dilutions or single concentrations of BV (Slade Health Pty Ltd), MMAE (MedChem Express) and tariquidar (Sigma-Aldrich) were added to growth media and incubated under normal culture conditions of 5% O_2_ for 72 h prior to assessment by resazurin assay. Responses were analysed by four-parameter logistic model by non-linear regression in GraphPad Prism 8.1.2 (GraphPad Software, La Jolla, CA, USA) to calculate the GI_50_ value for each cell type.

### In vivo drug efficacy studies in eIMS xenografts

Brentuximab doses were selected based on published xenograft lymphoma models^38–42^ and taking into account dose strategies used for consolidation therapy and treatment of refractory Hodgkin Lymphoma.^43,44^ Prior to undertaking drug treatment studies in tumour-bearing mice, treatment tolerability and toxicity studies were conducted in non-tumour-bearing NSG mice (2–4 mice per group). The crizotinib doses selected for the study in NSG mice have been published.^45^ Ceritinib doses had been previously tested for tolerability in NSG mice at doses ranging from 6.25 mg/kg, 12.5 mg/kg and 25 mg/kg. Brentuximab tolerability studies included twice-weekly dosing at 3 mg/kg, 6 mg/kg and 12 mg/kg for 6 weeks (12 doses in total). Combination schedules, including BV (1 mg/kg twice-weekly for 12 doses) combined with either crizotinib (50 mg/kg/day for 28 days) or ceritinib (25 mg/kg daily for 28 days) were performed for tolerability and toxicity prior to treatment of tumour-bearing mice. For tolerability studies, monitoring was performed twice weekly and mice were monitored throughout treatment and for 3 weeks after the cessation of treatment. Mice were euthanised at the end of the 3-week monitoring period. Drug treatments were commenced when xenografts reached a volume of 150 mm^3^. Power calculation based on the resource equation method indicated four to eight mice per treatment group.^46^ Mice were treated either with single-agent BV (1 mg/kg or 3 mg/kg, twice weekly for 6 weeks) delivered intravenously (IV) or combinations of BV and crizotinib (Jomar Life Research) (25, 50 or 100 mg/kg oral gavage daily for 28 days), ceritinib (Active Biochem) (6.25, 12.5 or 25 mg/kg oral gavage daily for 28 days), or tariquidar (12 mg/kg oral gavage given 1 h before each BV injection). Mice that showed no evidence of tumour regrowth were euthanised 170 days after completion of treatment and subjected to necropsy to confirm absence of tumour. Response to treatment was scored using criteria established by the Pediatric Preclinical Testing Consortium (PPTC).^47^ Mice that demonstrated less than 50% regression of the tumour volume at the start of treatment were defined as having progressive disease (PD). This category was further subdivided into PD1 or PD2 according to the tumour growth delay value, calculated by dividing the time to endpoint for each mouse by the median endpoint for the vehicle-control group. PD1 was assigned for a tumour growth delay value less than or equal to 1.5, and PD2 was defined by a tumour growth delay value greater than 1.5. A partial response (PR) was defined as tumour regression to a volume less than 50% of the starting, while a complete response (CR) was defined by disappearance of measurable tumour for at least one observation. If tumour volume was not measurable at the end of the study period, defined as 170 days after the last treatment, the response was considered maintained (MCR).^47^

### Statistical analyses

Curve fitting, analysis and data visualisation were performed using GraphPad Prism v8.1.2. Dose–response curves were fit using a four-parameter logistic model by non-linear regression, and GI_50_ calculated by interpolation. Statistical comparison of GI_50_ values, flow cytometry and western blot were performed by one-way ANOVA with Tukey multiple-comparisons test, with *P* < *0.05* considered statistically significant. For categorically scored CD30 expression by IHC, statistical comparison was performed by one-way ANOVA using the Kruskal–Wallis test with Dunn’s multiple-comparisons test. For in vivo studies, response to each drug treatment was assessed by Kaplan–Meier survival analysis with Mantel–Cox testing.

## Results

### Establishment and validation of diagnosis and relapse patient-derived eIMS xenografts

eIMS cell cultures were established from malignant ascites collected from a patient at diagnosis and following multiple, sequential lines of treatment, including crizotinib, ceritinib and chemotherapy (methotrexate, vinorelbine and ketorolac).^9,10^ The *RANBP2-ALK* translocation was detected in all samples collected from this patient, as reported.^9,10^ Previously, an *ALK*-resistance mutation (I1171T) had been identified in specimens collected following relapse on crizotinib and again following progression on ceritinib treatment.^9^ However, the I1171T *ALK* mutation was not found in eIMS cells collected at terminal relapse used to generate the relapse eIMS model. This sample was collected at the point of disease progression following treatment with low-dose chemotherapy, but without any ALK inhibitor treatment for over 6 months. A timeline of the patient’s treatment, response and collection of clinical samples is depicted in Fig. 1a.

Xenograft models were established in NSG mice by intraperitoneal inoculation of cultured patient-derived eIMS cells. Multinodular intra-abdominal disease, recapitulating the patient’s clinical presentation,^9^ developed in all mice inoculated with either the diagnosis (*n* = 2) (Fig. 1b) or relapse cells (*n* = 4) (Fig. 1c). The two mice inoculated with eIMS-diagnosis cells were euthanised for weight loss 102 and 116 days after inoculation, while the four mice inoculated with eIMS-relapse cells were euthanised for weight loss 47, 48, 49 and 53 days post-inoculation. The xenograft identity was validated against the patient germline by STR profiling (Table 1). Interphase FISH performed on xenograft tumour cells demonstrated an *ALK* translocation consistent with the original tumour (Fig. 1d, representative image from diagnosis xenograft). Histologic assessment of the xenografts confirmed features comparable to the original patient tumour; epithelioid morphology, ALK and CD30 staining (Fig. 1e). The *RANBP2-ALK* fusion identified in the patient tumour^9,10^ was confirmed by Sanger sequencing of both diagnosis and relapse xenograft material (Fig. 1f).^9,10^ There was no evidence of the I1171T *ALK* mutation in either the diagnosis or relapse xenografts by RNA Capture sequencing.^10^ There was no significant difference in the in vitro sensitivity to crizotinib or ceritinib between the diagnosis and relapse cells (Supplementary Fig. 1). Together these analyses showed the paired *RANBP2-ALK* eIMS xenograft models recapitulated the clinical, pathologic and molecular features of the patient.

### eIMS is sensitive to BV treatment in vitro and in vivo

**Fig. 2:**
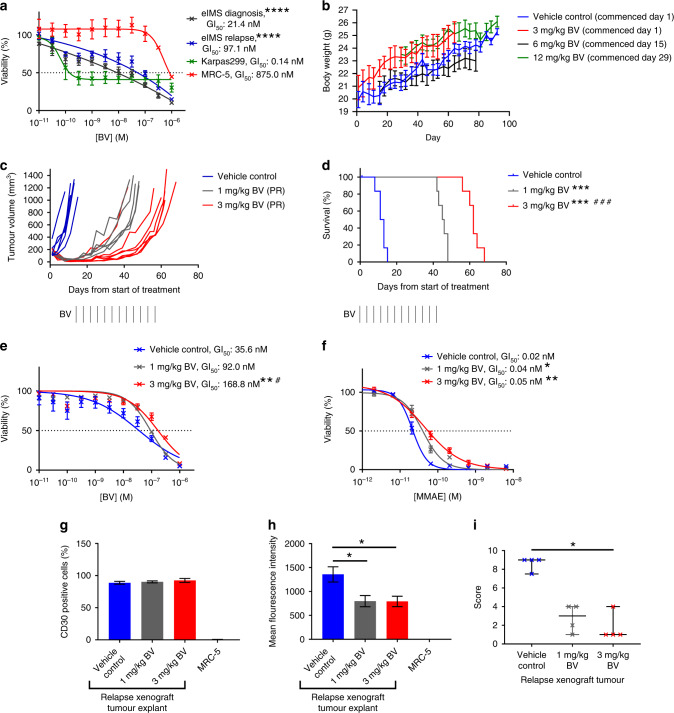
eIMS cells and xenografts are sensitive to BV; however resistance develops during the treatment period. **a** Resasurin cytotoxicity assay after 72 h BV treatment in eIMS-diagnosis and relapse cells (mean survival ± SEM from three independent assays, mean GI_50_ calculated by interpolation). Karpas299 was included as a positive control. ****: Comparison of eIMS to MRC-5, *P* < 0.0001 *(*one-way ANOVA with Tukey multiple-comparisons test). **b** Serial mouse weight (mean weight ± SEM)) during 6-week tolerability and toxicity studies of single-agent BV in non-tumour-bearing mice. **c** Tumour growth curves, PPTC objective response and **d** Kaplan–Meier survival of subcutaneous eIMS-relapse xenografts treated with 1 mg/kg or 3 mg/kg BV for six weeks (black vertical lines). ***: Comparison of BV treatment group to vehicle-control group, *P* < 0.001, and ###: comparison of BV treatment groups *P* < 0.001 *(*Mantel–Cox test). Resasurin cytotoxicity assay after 72 h of single cell suspensions of treated xenograft cells retreated with either **e** BV or **f** MMAE (mean viability ± SEM from three independent assays, mean GI_50_ calculated by interpolation). *: Comparison of BV treatment to vehicle control, *P < 0.05, **P < 0.01, and #: comparison of 3 mg/kg BV to 1 mg/kg BV, *P* < 0.05, (one-way ANOVA with Tukey multiple-comparisons test). **g**–**i** CD30 expression in treated xenografts. CD30 expression in early passage treated xenografts was quantified by flow cytometry to measure **g** the percentage of CD30-positive cells and **h** the mean fluorescent intensity of CD30 expression in vehicle control and BV-treated xenograft tumour cells (means ± SEM from three independent analyses). Xenograft cells were derived from two mice per treatment group. *: Comparison of BV treatment to vehicle control, *P* < *0.05, (*one-way ANOVA with Tukey multiple-comparisons test). **i** Quantification of CD30 expression by IHC in formalin fixed xenograft tumours. CD30 expression was quantified by blinded scoring of the relative proportion and intensity of CD30 staining in ten fields of view from one tumour section per mouse. Staining intensity values are mean score ± SEM from two mice. *: Comparison of BV treatment to vehicle control, *P* < 0.05*, (*one-way ANOVA with Kruskal–Wallis and Dunn’s multiple-comparisons test).

Having confirmed CD30 expression in the eIMS xenografts, in vitro sensitivity of eIMS cells to the CD30-targeted antibody–drug conjugate, BV, was evaluated. BV sensitivity of eIMS cells was compared to MRC-5 fibroblasts (CD30 negative, Supplementary Fig. 2) and Karpas299 lymphoma cells (CD30 positive^48^) as negative and positive controls, respectively. eIMS-diagnosis and relapse cells were significantly more sensitive to BV than MRC-5 cells with GI_50_ values of 21.4, 97.1 and 875.0 nM respectively (*P* < 0.0001 for both comparisons) (Fig. 2a).

To determine whether BV might be useful for the treatment of eIMS, the in vivo efficacy of BV was evaluated using the relapse eIMS subcutaneous xenograft model. Prior to testing BV against the relapse eIMS model, single-agent BV tolerability studies were carried out in non-tumour-bearing mice. Non-tumour-bearing mice were treated with BV twice per week for 6 weeks. We did not observe toxicity at any dose level tested over 6 weeks (Fig. 2b). The relapse eIMS xenograft was inoculated into immunodeficient mice and once tumours reached 150 mm^3^, treatment was initiated at either 1 mg/kg (*n* = 6) or 3 mg/kg BV (*n* = 6), administered  twice per week for 6 weeks. Initial tumour regression was observed in all mice at both doses of BV (Fig. 2c) and was scored as a PR by objective-response criteria.^47^ Mice treated with 1 mg/kg BV experienced a median survival of 45.5 days (range 42.0–48.0 days), which was significantly longer than mice that received vehicle control (*n* = 6), with median survival of 12.0 days (range 8.0–15.0 days) (*P* < 0.001). Furthermore, survival significantly improved with 3 mg/kg treatment compared to 1 mg/kg treatment (Fig. 2d), with survival extended to a median of 62.0 days (range 56.0–68.0 days) (*P* < 0.01). Treatment with 3 mg/kg BV also resulted in significant survival extension compared to the vehicle-control group (*P* < 0.01). However, tumours regrew during BV treatment at both doses (Fig. 2c). Thus, although BV extended survival in the eIMS-relapse xenograft model, regrowth during treatment suggests that BV is unlikely to provide durable tumour control in patients when used as a single agent.

### BV-treated eIMS tumours develop resistance to BV

To determine whether regrowth of the eIMS-relapse xenograft tumours during BV treatment in vivo correlated with acquired resistance to BV, in vitro sensitivity to BV was assessed using cells isolated from treated xenograft tumours. In vitro growth-inhibition assays confirmed that tumour cells from 3 mg/kg BV-treated mice were more resistant to BV (GI_50_: 168.8 ± 1.2 nM) compared with tumour cells from mice treated with vehicle control (GI_50_: 35.6 ± 1.4 nM) (*P* < 0.01) (Fig. 2e). Tumour cells from mice treated with 3 mg/kg BV were also significantly more resistant to BV compared with cells from xenograft mice that received 1 mg/kg BV (GI_50_: 92.0 ± 1.1) (*P* < 0.05).

### BV-treated eIMS tumours develop resistance to MMAE

To determine whether BV resistance in the eIMS-relapse xenograft was attributable to reduced sensitivity to the active chemotherapeutic component of BV, MMAE, the sensitivity of BV-treated xenograft tumour cells to MMAE was assessed (Fig. 2f). In an in vitro growth-inhibition assay, xenograft cells derived from tumours that acquired resistance to BV in vivo were found to be significantly more resistant to MMAE (1 mg/kg-treated xenograft tumour cells: GI_50_ 0.04 ± 0.009 nM, 3 mg/kg-treated xenograft tumour cells: 0.05 ± 0.024 nM) compared to xenograft tumour cells from vehicle-control-treated mice (GI_50_: 0.02 ± 0.004 nM) (*P* < 0.05 for both comparisons). These data suggest that acquired MMAE resistance contributed to the development of resistance to BV in vivo.

### CD30 is downregulated in BV-treated eIMS tumours

To determine if downregulation of CD30 expression may have contributed to BV resistance in vivo, CD30 expression was examined in xenograft tumour samples by flow cytometry and IHC. The percentage of CD30 positive cells in dissociated tumour was not reduced by BV treatment (Fig. 2g). However, flow cytometry showed a significant reduction in the CD30 mean fluorescence intensity in BV-treated tumours; 797.6 ± 117.3 for 1 mg/kg and 792.2 ± 108.8 for 3 mg/kg BV-treated tumours, compared to 1357.0 ± 159.3 in vehicle control treated mice (*P* < 0.05 for both comparisons) (Fig. 2h). Semi-quantitative scoring of CD30 expression in xenograft tumours by IHC showed a reduction in CD30 stain intensity from a median score of 9.0 (range 7.5–9.0) for vehicle control treated mice, to 3.0 (range 1.0–4.0) for 1 mg/kg BV-treated mice and 1.0 (range 1.0–4.0) for 3 mg/kg BV-treated mice compared to vehicle control (*P* < 0.05, for comparison of control vs 3 mg/kg treatment) (Fig. 2i). Collectively, these data suggest that reduced CD30 expression, rather than loss of CD30 positive cells, may have contributed to BV resistance in vivo.

### ABCB1 is upregulated in BV-treated eIMS tumours

**Fig. 3:**
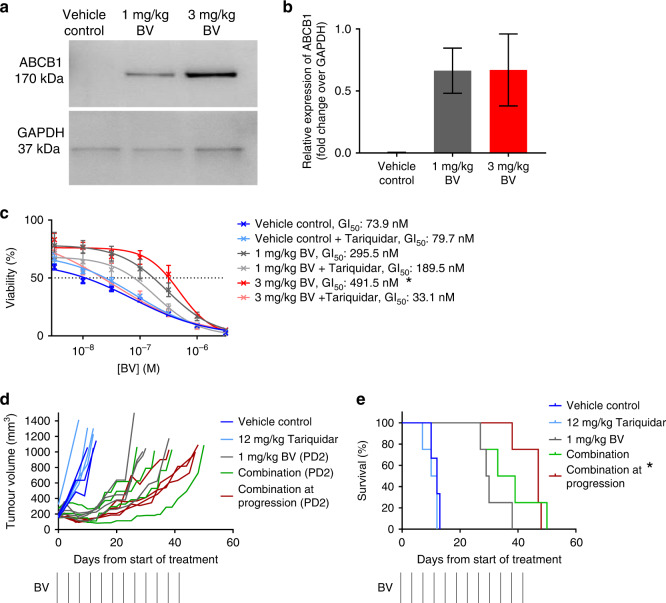
ABCB1 is upregulated in relapse eIMS xenografts following BV treatment. ABCB1 expression in BV treated eIMS xenografts. Protein was extracted from treated xenograft tumours and ABCB1 expression was analysed by **a** western blot and **b** quantified using GAPDH as a loading control (mean ± SEM from three independent blots). **c** Tariquidar treatment restores in vitro BV sensitivity. Resasurin cytotoxicity assay after 72 h treatment in vitro with BV ± 100 nM tariquidar of explanted xenografts (mean viability ± SEM from three independent assays, mean GI_50_ calculated by interpolation). *: Comparison of BV treatment to vehicle control, *P* < 0.05*, (*one-way ANOVA with Tukey multiple-comparisons test). **d** Tumour growth, PPTC objective response and **e** Kaplan–Meier survival of the relapse eIMS xenograft treated with BV ± tariquidar. Relapse eIMS xenografts were established and mice were treated with vehicle control, tariquidar, single-agent BV or tariquidar and BV. For the single-agent BV treatment group, once tumours reached 150 mm^3^ in size, mice were randomised to continue treatment with either with single-agent BV, or the combination of tariquidar and BV. *: Comparison of combination to 1 mg/kg BV, *P* < 0.05, (Mantel–Cox test).

ABCB1 was previously implicated in BV resistance due to MMAE efflux in Hodgkin's Lymphoma.^49^ ABCB1 expression was evaluated in tumour samples from BV-treated mice by western blot. These analyses showed that ABCB1 was upregulated in BV-treated tumours (Fig. 3a, b), with expression detected in both 1 mg/kg and 3 mg/kg BV-treated tumours, but undetectable in vehicle-control-treated tumours.

### ABCB1 inhibition with tariquidar restored BV sensitivity in resistant tumour cells

We next examined whether pharmacological inhibition of ABCB1 could restore BV sensitivity in the resistant xenograft eIMS cells using in vitro growth-inhibition assays. Tariquidar (100 nM) treatment of tumour cells from mice treated with 3 mg/kg BV resulted in a 14.8-fold sensitisation to BV (*P* < 0.01) to a give a GI_50_ value that was not significantly different from BV sensitive control cells (Fig. 3c). Tariquidar treatment of tumour cells from mice treated 1 mg/kg BV resulted in more modest sensitisation to BV, although this change did not reach statistical significance. Therefore, tariquidar was able to restore BV sensitivity in resistant cells from mice treated with 3 mg/kg BV.

### ABCB1 inhibition with tariquidar enhances the efficacy of BV in mice harbouring eIMS-relapse xenografts

We next tested the hypothesis that the survival of mice engrafted with eIMS-relapse tumours would be prolonged by treatment with tariquidar in combination with BV compared to treatment with BV alone. In this experiment, relapse eIMS xenograft mice were treated with vehicle control (three mice), single agent 12 mg/kg tariquidar twice weekly for 6 weeks (four mice), single agent 1 mg/kg BV twice weekly for 6 weeks (eight mice) or combination therapy with tariquidar and BV (four mice). Treatment with 1 mg/kg BV led to tumour regression, followed by subsequent regrowth during the BV treatment period, as previously observed (Fig. 2b). When tumour regrowth reached 150 mm^3^, BV-treated mice were randomised to either continue single-agent BV (four mice), or to receive tariquidar co-administered with the remaining scheduled doses of BV (four mice). There was no difference in the survival of mice treated with tariquidar or vehicle control. Combination treatment with tariquidar and BV did not influence tumour growth (Fig. 3d) or significantly improve survival compared with single-agent BV (Fig. 3e). At the point of tumour progression following single-agent BV treatment, addition of tariquidar slowed the rate of tumour growth compared to mice which were continued on BV alone (Fig. 3d), although no tumour regression occurred after the introduction of the combination treatment (Fig. 3d). The median survival of mice treated with BV and tariquidar (after progression on BV alone) was extended to 47.0 days (range 38.0–48.0 days) compared with mice that received BV alone, which had a median survival of 29.5 days (range 27.0–38.0 days) (*P* < 0.05) (Fig. 3e). Mice on all therapy schedules experienced PD2 by objective-response criteria.^47^

### Combination therapy for eIMS

**Fig. 4:**
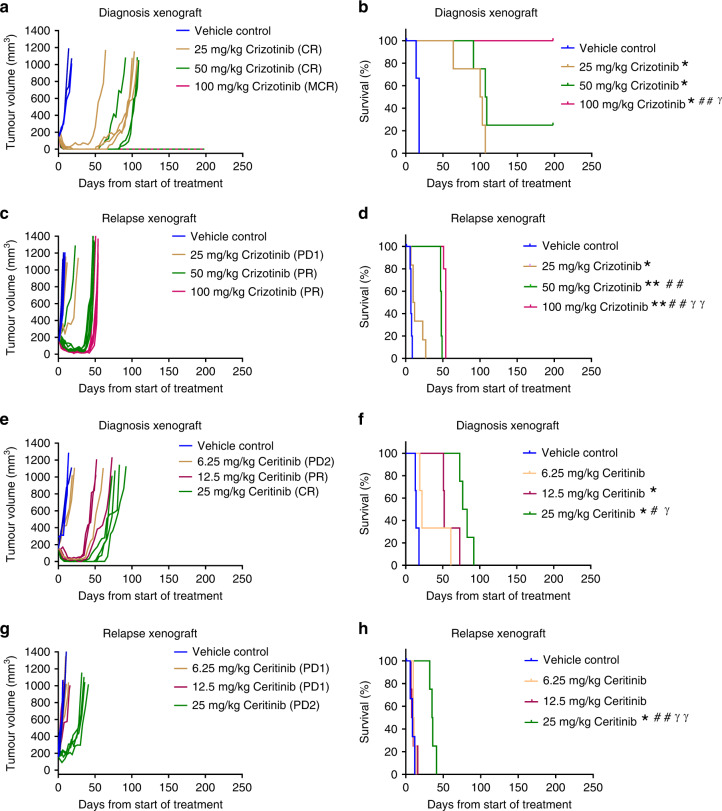
Single-agent dose–response for ALKi in diagnosis and relapse eIMS xenografts. Tumour growth curves, PPTC objective response and Kaplan–Meier survival analysis of diagnosis and relapse eIMS xenografts treated with **a**–**d** crizotinib or **e**–**h** ceritinib. Vehicle-control groups are three to six mice and treatment groups are four to six mice. *: Comparison of treatment group to vehicle-control group. #: Comparison to lowest dose treatment group. γ: Comparison of highest dose treatment group to middle dose treatment group. *P < 0.05, **P < 0.01, (Mantel–Cox Test).

We next examined the efficacy of BV in combination with the ALKi, crizotinib, in the diagnosis and relapse settings. In vitro studies performed on eIMS-diagnosis and relapse cells suggested that the combination of BV and crizotinib may be more potent than single agent treatment. Synergy or additivity was observed when the eIMS-diagnosis cells were treated with 31.6 nM crizotinib and BV at concentrations ranging from 0.3–10.0 nM (Supplementary Fig. 3A). In contrast, no evidence of synergy was seen in the eIMS-relapse cells (Supplementary Fig. 3B). To determine a suitable dose of crizotinib to use in combination with BV in vivo, single agent dose–response was evaluated in the diagnosis (Fig. 4a, b, *n* = 4 mice per group) and relapse (Fig. 4c, d, *n* = 5 mice per group) eIMS xenografts. As 100 mg/kg crizotinib led to an MCR in the diagnosis xenograft, a lower dose of 50 mg/kg crizotinib was chosen for combination testing. In both the diagnosis and relapse xenografts, a 50 mg/kg dose demonstrated a significant median survival extension compared with the vehicle-control groups (*P* < 0.05), without achieving an MCR in the majority of mice (Fig. 4a–d).

Similar studies performed on eIMS-diagnosis and relapse cells in vitro demonstrated that the combination of BV and ceritinib enhanced growth inhibition compared to single agent treatment. Additivity was observed for all combinations of BV and ceritinib examined in the eIMS-diagnosis and -relapse cells (Supplementary Fig. 3C and 3D). To determine a suitable dose of ceritinib to use in combination with BV, single agent dose–response was evaluated in the diagnosis and relapse eIMS xenografts (Fig. 4e–h, *n* = 4 mice per group). A dose of 25 mg/kg ceritinib was chosen for combination testing as this dose demonstrated a significant median survival extension compared to the vehicle-control groups (*P* < 0.05) without achieving an MCR. Selection of these single agent doses allowed room for evaluation of combination efficacy relative to both single agent and vehicle-control groups. Notably, tumour-free survival was prolonged by treatment with crizotinib or ceritinib for diagnosis eIMS mice when compared to relapse eIMS mice which were administered the same treatment schedule. Prior to testing BV and ALK inhibitor combinations against the eIMS xenografts, combination studies were performed in non-tumour-bearing mice to confirm tolerability of each combination. In comparison to vehicle control, no toxicity was observed in mice treated the crizotinib (50 mg/kg for 28 days) and BV (1 mg/kg twice weekly for 6 weeks) nor in mice treated with ceritinib (25 mg/kg for 28 days) and BV (1 mg/kg twice weekly) (Supplementary Fig. 3E and F).

**Fig. 5:**
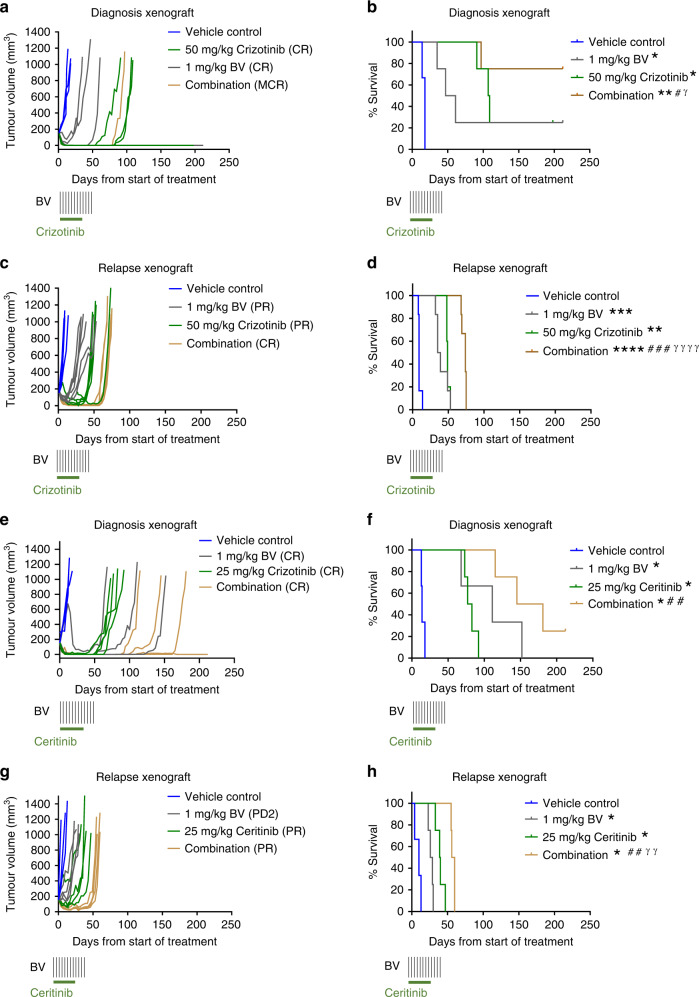
Combination of BV with ALKi is more effective than single agent therapy for eIMS. Tumour growth, PPTC objective response and Kaplan–Meier survival curves for **a**, **b** diagnosis or **c**, **d** relapse eIMS xenografts treated with BV (twice weekly for six weeks IV, indicated by black vertical lines), crizotinib (daily for 28 days PO, indicated by horizontal green line) or combination, and **e**, **f** diagnosis or **g**, **h** relapse eIMS xenografts treated with BV (twice weekly for six weeks IV, indicated by black vertical lines), ceritinib (daily for 28 days PO, indicated by horizontal green line) or combination. Vehicle-control groups are three mice, treatment groups are four mice. *: Comparison of treatment group to vehicle-control group. #: Comparison of combination treatment group to 1 mg/kg BV treatment group. γ: Comparison of combination treatment group to ALKi treatment group. *P < 0.05, **P < 0.01, ***P < 0.001, **** P < 0.0001 (Mantel–Cox Test). Vehicle control and single agent ALKi groups for the diagnosis xenografts are the same as presented in the combination studies in Fig. 4.

During in vivo efficacy studies of the BV and crizotinib combination, tumour regression was observed in all mice harbouring the diagnosis eIMS xenograft (*n* = 4 mice per group) (Fig. 5a). Three of four mice treated with crizotinib plus BV showed no evidence of tumour at 212 days, 170 days after the end of the 42-day treatment. All three mice were confirmed tumour-free upon necropsy. This was a significant survival advantage for combination therapy over either single agent crizotinib (median survival 108 days, range 91–212 days) or BV (median survival 61 days, range 35–212 days) (*P* < 0.05 for both comparisons) (Fig. 5b). By objective-response criteria, three out of four mice treated with the crizotinib and BV combination achieved an MCR, while one mouse achieved a CR. Mice on single-agent BV or crizotinib demonstrated CR, with only one mouse in each group achieving an MCR (Fig. 5a).

When tested against the relapse eIMS xenograft, the combination of BV and crizotinib was also more effective than either BV or crizotinib alone (*n* = 4–6 mice per group) (Fig. 5c). Combination therapy prolonged the survival of xenografted mice to a median survival of 74 days (range 68–75 days) compared to 37 days (range 32–53 days) with BV alone (*P* < 0.001). Survival was also significantly extended by combination treatment compared with the crizotinib-only treatment group, with a median survival of 48.5 days (range 48–53 days) (Fig. 5d) (*P* < 0.0001). According to objective-response criteria, five of six mice with relapse eIMS xenografts showed a CR following combination treatment, while mice treated with single agents demonstrated only PR or PD2 (Fig. 5c). In summary, a combination of BV and crizotinib treatment rendered 3 of 4 mice bearing the diagnosis eIMS PDX tumour free after >180 days of observation. In contrast, whilst the BV and crizotinib treatment significantly prolonged the survival of mice bearing the relapse of eIMS PDX, all mice experienced disease progression within 100 days.

When testing the combination of BV and ceritinib, tumour regression was observed in all mice harbouring the diagnosis eIMS xenograft (*n* = 4 mice per group) (Fig. 5e). This extended survival significantly to 163 days (range: 115–212) compared to single agent ceritinib (median survival 80 days, range 73–92 days) (*P* < 0.01), but not single-agent BV (median survival 111 days, range 68–152 days) (Fig. 5f). By objective-response criteria, mice treated with the ceritinib and BV combination achieved a CR or MCR. Mice on single-agent BV or ceritinib demonstrated PR or CR (Fig. 5e).

The combination of BV and ceritinib in the eIMS-relapse xenograft was more effective than either BV or ceritinib alone, with tumour regression observed in all mice (*n* = 4 mice per group) (Fig. 5g). Combination therapy prolonged survival of xenografted mice to a median survival of 58 days (range 55–60 days) compared to 27.5 days (range 23–30 days) with BV alone (*P* < 0.01). Survival was also significantly extended by combination treatment compared to the ceritinib only treatment group, with a median survival of 39.5 days (range 33–47 days) (Fig. 5h) (*P* < 0.01). According to objective-response criteria, all combination treated mice achieved a PR, while mice treated with single agents demonstrated only a PR or PD2.

## Discussion

eIMS is an aggressive subtype of IMT which requires new treatment options.^8,25^ Our recent retrospective cohort study showed that patients with *RANBP2-ALK*-rearranged eIMS-relapsed despite initial complete responses to crizotinib.^10^ Previous reports have also shown poor response to various treatments, including chemotherapy and radiotherapy in eIMS patients.^8–10^ Prior to the introduction of ALK inhibitors, most patients experienced rapid disease progression and died despite treatment with chemotherapy and/or radiation therapy.^8,20^ Prior to the current study, there were no appropriate models available for investigating new therapies for eIMS. The matched diagnosis and relapse cell lines and xenografts generated in this study are the first reported patient-derived xenograft eIMS models and have allowed preclinical investigations of new treatment options to be conducted specifically for this disease. Patient-derived xenograft models of solid tumours have been shown to accurately reflect patients’ response to therapy, and are considered a useful tool in the assessment of novel therapeutic options.^50,51^ The eIMS xenograft models are of value as they represent treatment naïve and relapsed disease following ALKi and chemotherapy, facilitating evaluation of novel therapeutic options applicable to patients, either in front line therapy or for treatment after relapse.

An ALKi-resistance mutation (I1171T) had been identified in eIMS tumour samples collected following a relapse on crizotinib and again following disease progression during ceritinib treatment.^9^ However, the resistance mutation was not detected in the eIMS tumour sample which was collected at terminal relapse after prolonged chemotherapy treatment but without an ALK inhibitor. Although unexpected, loss of a crizotinib-resistance mutation is not unprecedented.^52^ Michels et al described a patient with *ROS1*-rearranged lung cancer who developed progressive disease during crizotinib therapy, following which an exon 38 *ROS1* resistance mutation (G2032R) was identified.^52^ The crizotinib was ceased and the tumour responded to chemotherapy. The patient was observed until further disease progression approximately two years later. Resequencing of a repeat tumour biopsy showed only wild type *ROS1* sequence in the recurrent tumour, with no evidence of the G2032R mutation. The tumour responded to retreatment with crizotinib with evidence of an ongoing partial response 12 months later.^52^

We have previously published the clinical response and outcome following crizotinib and ceritinib treatment for the patient from which the *RANBP2-ALK*-rearranged patient-derived models have been generated.^9,10^ Although important to document on target activity for novel drug-target combinations, the ALK inhibitors, crizotinib and ceritinib, have undergone extensive preclinical characterisation including confirmation of on target activity in ALK-rearranged models.^24,53,54^ Since the publication of clinical trials conducted by the COG and EORTC, ALKi treatment is regarded as a standard of care for unresectable and/or multifocal *ALK*-rearranged IMT.^12–14^ In this context, we have focused our PDX experiments on treatment response and survival based on the measures developed by the NCI Pediatric Preclinical Testing Consortium to prioritise treatments for clinical trials.^47^ CD30 is a previously unexplored therapeutic target in eIMS. The CD30-targeted antibody–drug conjugate, BV, has undergone extensive preclinical and clinical evaluation, has been approved by multiple regulatory agencies, including both the FDA and the EMA, for the treatment of Hodgkin Lymphoma, ALCL and CD30 positive cutaneous T-cell lymphoma and is currently in use in 40 clinical trials for children and teenagers as listed in ClinicalTrials.gov (https://www.clinicaltrials.gov/ct2/results?term=brentuximab&Search=Apply&age_v=&age=0&gndr=&type=&rslt=).^29–31^ The present study was designed to examine the in vivo efficacy of BV against eIMS xenografts. BV does not bind mouse CD30 and our studies were not designed to examine BV tolerability and toxicity which has been previously examined. We adapted BV doses and schedules from prior publications,^38–41^ and taking into account doses used in clinical trials of relapsed Hodgkin lymphoma^43,44^ and did not observe unexpected toxicity either from BV as a single agent nor when used in combination. We have shown that BV delivered as a single agent induced growth inhibition of eIMS cells in vitro, as well as reduced tumour burden and prolonged survival in the diagnosis and relapse eIMS xenograft models. However, a complete in vivo response to BV as a single agent was not achieved as tumours regrew while therapy was being administered. Flow cytometry and IHC analysis showed that CD30 antigen expression was reduced following BV treatment, which may have limited the binding and/or uptake of BV. Consistent with findings in the current study, a reduction in CD30 expression was previously observed in BV-resistant Karpas299 and KM-H2 cell lines that were generated by prolonged exposure to BV in vitro.^49,55^ Increased expression of the multidrug transporter ABCB1 was also identified as a possible BV-resistance mechanism in the eIMS xenografts, consistent with a previous report showing efflux of MMAE via ABCB1 in a Hodgkin's Lymphoma cell line.^49^ We have not experimentally defined mechanism(s) underlying reduced cell surface CD30 expression following BV treatment, which may include the selection and expansion of a sub-clonal population with intrinsically lower CD30 expression, downregulation of CD30 expression, increased turnover of CD30 or as suggested by Chen and colleagues altered dynamics in CD30 internalisation reducing cell surface CD30 expression.^49^ Further investigations are warranted to determine whether BV resistance resulted from inhibition of CD30 expression and upregulation of ABCB1 during treatment, or to expansion of a pre-existing subclone with inherently low CD30 expression and/or high ABCB1 expression.

Progressive tumour regrowth during BV therapy in both the diagnosis and relapse eIMS xenografts suggests there is limited clinical potential for BV as a single agent treatment for eIMS. While ABCB1 inhibition re-sensitised BV-treated xenograft cell cultures to BV in vitro, only a modest improvement in the survival of BV-treated xenograft mice was observed after combination treatment with the ABCB1 inhibitor tariquidar in vivo. These results suggest that whilst ABCB1 inhibition may extend the effective treatment window of BV in eIMS patients, the overall clinical impact may be minimal. The combination of BV with the first-generation ABCB1 inhibitor verapamil is presently being explored in CD30-positive lymphoma (clinical trial NCT03013933).^42^ However, this is the first preclinical study of the third-generation inhibitor tariquidar in eIMS. While application of the combination of BV and tariquidar is possible in the clinic, the modest response to tariquidar observed in the eIMS models suggests that alternative combination strategies for treatment of this cancer should be pursued.

The ALK inhibitors, crizotinib and ceritinib, are in current clinical use for ALK-positive IMT.^10,12,20,56^ Therefore, the combinations of BV with crizotinib or ceritinib were evaluated based on the potential for rapid clinical translation as they have undergone early phase clinical trials in children and recommended phase 2 doses have been determined.^12,13^ In the diagnosis eIMS model, the combination of BV and crizotinib was highly efficacious, with all mice experiencing a CR and most animals confirmed as tumour-free at the end of the study (≥180 days post treatment). In contrast, despite initial complete responses, tumour recurrence within 100 days occurred in all relapse eIMS xenograft mice receiving BV and crizotinib. In both diagnosis and relapse xenograft models, the combination of BV and crizotinib was more effective than either single agent. Furthermore, the combination of BV and ceritinib was more effective in the eIMS-diagnosis model compared to BV alone, while in the eIMS-relapse xenograft the combination was more efficacious than either single agent treatment. Collectively, the xenograft data provides a strong rationale to use crizotinib and BV as combination therapy as a first-line treatment for eIMS. The combination therapy may also be effective in the treatment of relapse disease; however, this would need to be assessed in the context of the presence of ALKi-resistance mutations. The combination of BV with ceritinib is also a potentially useful combination in the treatment of eIMS. Although not directly examined in our work, BV and ALKi combination therapy may also be clinically applicable in other CD30-positive, ALK-rearranged malignancies such as ALCL. Wang and colleagues have recently demonstrated that the combination of crizotinib and an alternative CD30 antibody–drug conjugate, anti-CD30-lidamycin, were more effective than single agent therapy in both in vitro and xenograft models of ALCL.^57^

Our recent retrospective cohort study demonstrated that eIMS patients are at risk of early treatment failure despite early and complete responses to crizotinib.^10^ The present study demonstrates the potential for therapeutic exploitation of CD30 in eIMS. The results suggest that combination therapy targeting both CD30 and ALK at diagnosis would be more efficacious than single agent therapy or using combination therapy at the point of relapse. Comprehensive pathologic and genetic profiling of IMT at diagnosis to evaluate CD30 expression and define the *ALK* fusion may identify patients who would potentially benefit from treatment with BV and crizotinib. Whilst perinuclear accentuation of ALK staining and CD30 expression are characteristic features of eIMS,^8,10,27,58,59^ it is not clear whether CD30 expression is restricted to eIMS or is found in other molecular subtypes of IMT. IHC for CD30 may not be routinely performed in IMT diagnostic pathology, and, recently conducted clinical trials have not prospectively subtyped ALK-positive IMTs on ALK subcellular location, CD30 expression and *ALK*-fusion partner.^12–14^ We suggest that the evaluation of IMT should include CD30 expression, ALK staining pattern, molecular screening for cryptic *ALK* fusions and mutations, characterisation of *ALK*-fusion partners and identification of other targetable fusions in ALK-negative cases.

In summary, this study describes the generation of the first eIMS patient-derived cell cultures and xenografts and their application in preclinical evaluation of novel drug combinations for the treatment of eIMS. The results functionally validate CD30 as a therapeutic target in eIMS, showing the CD30-directed antibody–drug conjugate, BV, has efficacy as a single agent and in combination with ALKi. The results showing that survival outcomes are more favourable following crizotinib and BV combination therapy in the diagnosis model compared to the relapse model suggest that combination therapy from diagnosis rather than at the point of relapse would be more likely to confer long-term disease control. Finally, this study provides a strong rationale for initiating a clinical trial of ALKi in combination with BV in eIMS and potentially other ALK-positive IMTs with CD30 expression.

## Supplementary information

Supplementary Figures

## Data Availability

The data that support the findings of this study are available from the corresponding author upon reasonable request.
