# Supplementary material for: CD30 and ALK combination therapy has high therapeutic potency in RANBP2-ALK-rearranged epithelioid inflammatory myofibroblastic sarcoma
Source: Br J Cancer. 2020 Jul 20;123(7):1101–13. doi: 10.1038/s41416-020-0996-2 (PMC7524717; doi:10.1038/s41416-020-0996-2)
Supplement: Supplementary file 1 — Supplementary Figures [file 41416_2020_996_MOESM1_ESM.docx]

**
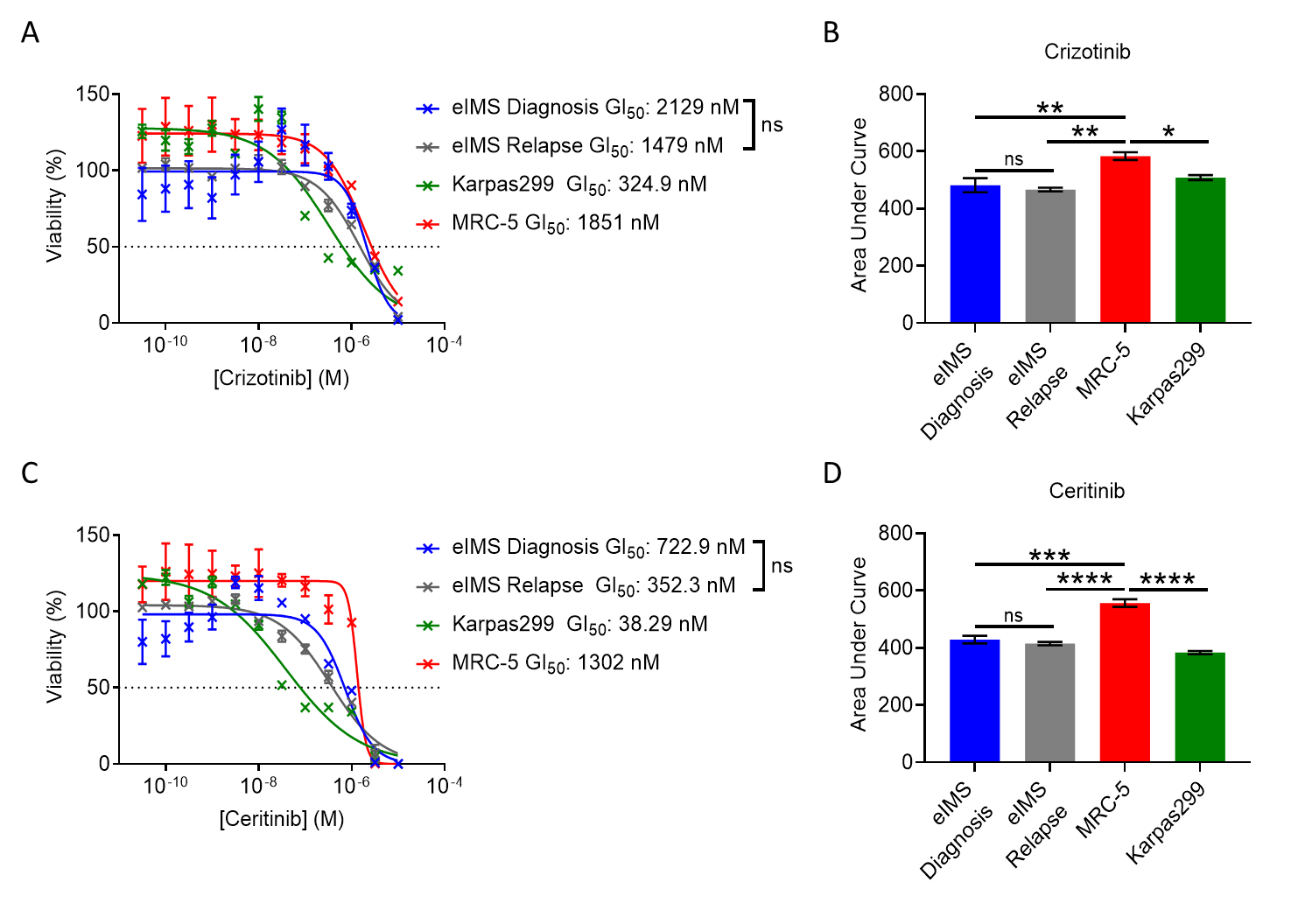
**

**Supplementary Figure 1.** eIMS diagnosis and relapse cells do not show differential response to *in vitro* treatment with crizotinib or ceritinib using the Cell Titre Glo® 2.0 assay. Cells were seeded in 384 well white-walled, clear bottom plates and incubated for 24 h after seeding before the addition of (A) crizotinib or (C) ceritinib. The CellTiter-Glo 2.0® reagent was used to evaluate growth inhibition after 72 h of ALK inhibitor treatment. Data is represented as mean percentage viability at each concentration from three independent experiments ± SEM. GI50 values were calculated using GraphPad Prism and annotated in the figure key. Statistical analysis of GI50 values was performed by one-way ANOVA with Tukey’s multiple comparisons test. Area under the curve ± SEM was calculated for the (B) crizotinib and (D) ceritinib dose response curves using GraphPad Prism. Statistical analysis was performed by one-way ANOVA with Tukey’s multiple comparisons test. ns: not significant. * *P < 0.05*, ** *P < 0.01,* *** *P < 0.001*, **** *P < 0.0001.*


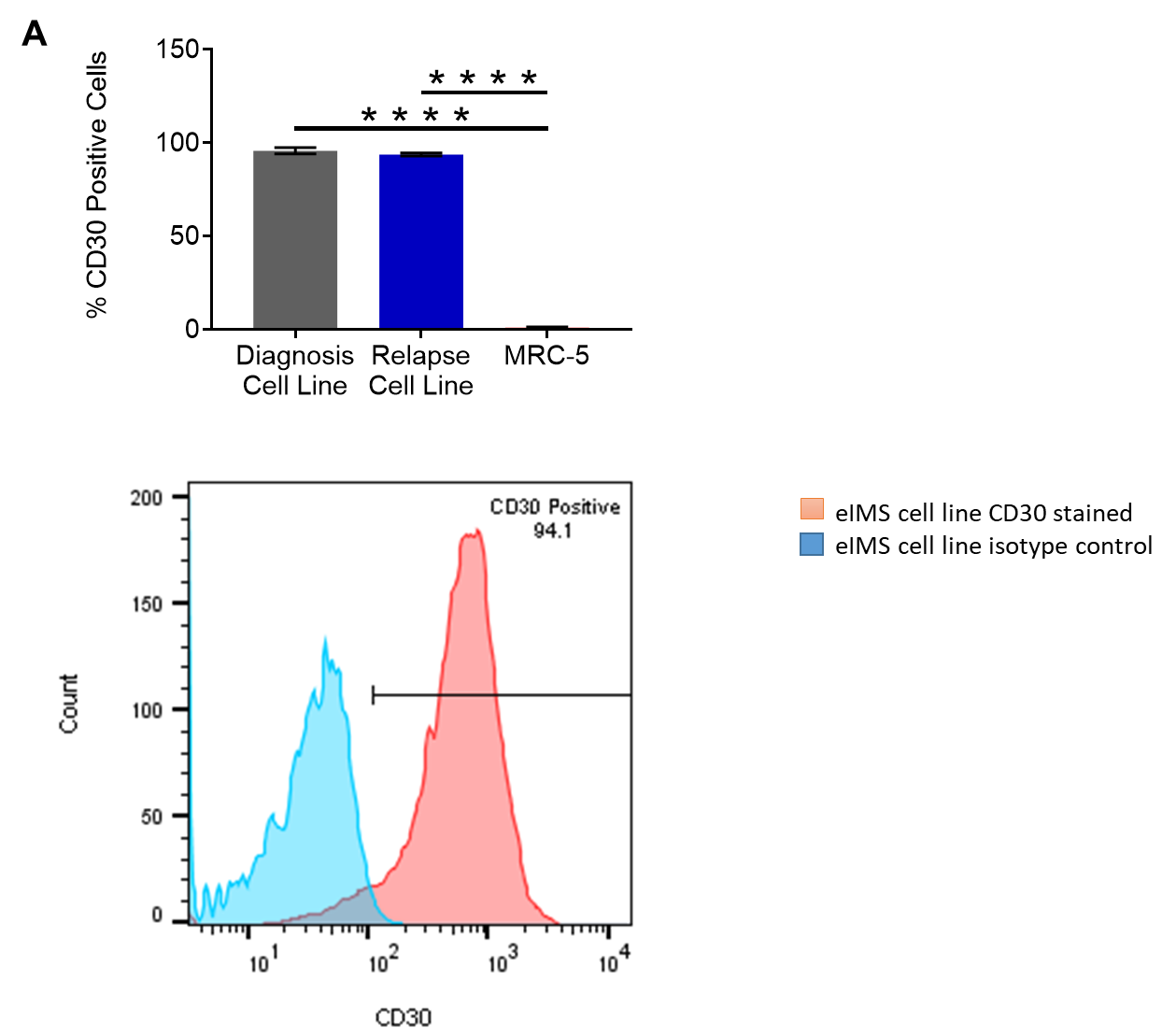


**Supplementary Figure 2.** **eIMS patient-derived cells are CD30 positive by flow cytometry.** For flow cytometric analysis, 1 × 10^5^ cells were harvested and stained for CD30 using PE-conjugated CD30 antibody (BD catalogue number 550041) or matched PE-conjugated mouse isotype control antibody (Ms IgG1K PE). (A) The percentage of CD30-PE positive cells in eIMS diagnosis, eIMS relapse and MRC-5 cell cultures was calculated from three independent experiments. Data is presented as mean percentage positive cells ± SEM. Statistical analysis was performed by one-way ANOVA with Tukey’s multiple comparisons test. ****: *P < 0.0001.* (B) Representative flow cytometry histogram plot of eIMS cells stained with CD30-PE (red) and isotype control antibody (blue).

**
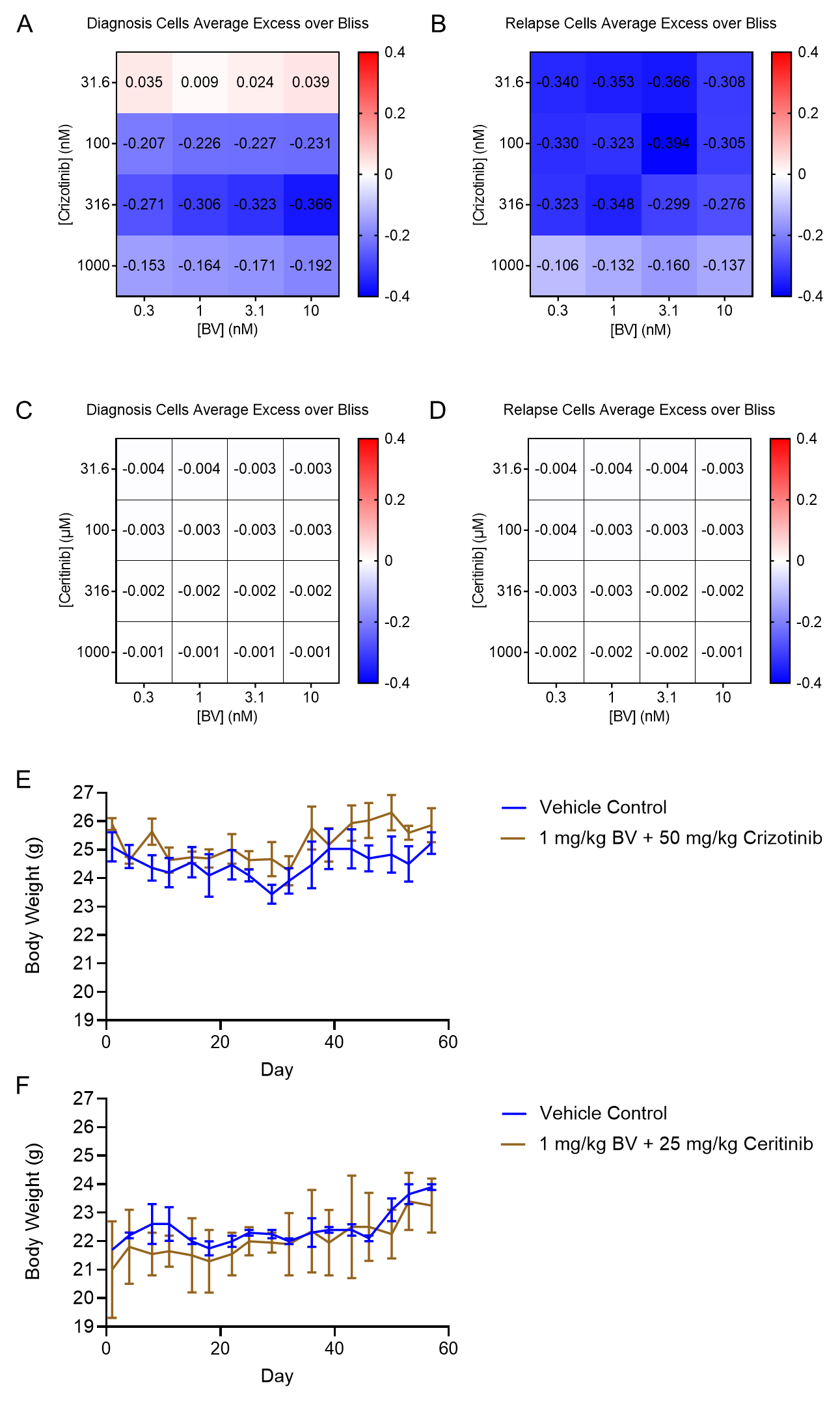
**

**Supplementary Figure 3: *In vitro* matrix screening of crizotinib plus brentuximab vedotin and ceritinib plus brentuximab vedotin and combination therapy tolerability studies in non-tumour bearing mice.** eIMS diagnosis (A and C) and relapse cells (B and D) were seeded at 2000 cells per well in 96 well plates. Cells were incubated according to normal culture conditions for 24 h after seeding. At 24 h, the agents were added such that crizotinib (A and B) and ceritinib (C and D) were tested in combination with BV, in a multiple point dose range. The assay was then measured at 72 hours with resazurin. Synergy was calculated using the excess over Bliss method, with the average across three experiments shown in the heatmap. Values below zero indicate antagonism, while values greater than zero suggest synergy. (E and F) Non-tumour bearing NSG mice were treated with combination therapy and monitored twice per week for 3 weeks after completion of treatment. (E) Mean body weight ± SEM of mice treated with BV (1mg/kg twice weekly for 12 doses) and crizotinib (50mg/kg/day for 28 days). (F) Mean body weight ± SEM of mice treated with BV (1mg/kg twice weekly for 12 doses) and ceritinib (25mg/kg/day for 28 days).
